# *Trypanosoma cruzi* interaction with host tissues modulate the composition of large extracellular vesicles

**DOI:** 10.1038/s41598-024-55302-3

**Published:** 2024-02-29

**Authors:** Izadora Volpato Rossi, Rafael Fogaça de Almeida, Bruna Sabatke, Lyris Martins Franco de Godoy, Marcel Ivan Ramirez

**Affiliations:** 1 Programa de Pós-graduação em Microbiologia, Parasitologia e Patologia, Universidade Federal do Paraná, Curitiba, Paraná Brazil; 2 EVAHPI Research Group, Laboratório de Biologia Celular, Instituto Carlos Chagas, Fundação Oswaldo Cruz, Curitiba, Paraná Brazil; 3 Laboratório de Biologia Molecular e Sistêmica de Tripanossomatídeos, Instituto Carlos Chagas, Fundação Oswaldo Cruz, Curitiba, Paraná Brazil

**Keywords:** *Trypanosoma cruzi*, Extracellular vesicles, Mass spectrometry, Microvesicles, Chagas disease, Cell biology, Microbiology, Pathogenesis

## Abstract

*Trypanosoma cruzi* is the protozoan that causes Chagas disease (CD), an endemic parasitosis in Latin America distributed around the globe. If CD is not treated in acute phase, the parasite remains silent for years in the host's tissues in a chronic form, which may progress to cardiac, digestive or neurological manifestations. Recently, studies indicated that the gastrointestinal tract represents an important reservoir for *T. cruzi* in the chronic phase. During interaction *T. cruzi* and host cells release extracellular vesicles (EVs) that modulates the immune system and infection, but the dynamics of secretion of host and parasite molecules through these EVs is not understood. Now, we used two cell lines: mouse myoblast cell line C2C12, and human intestinal epithelial cell line Caco-2to simulate the environments found by the parasite in the host. We isolated large EVs (LEVs) from the interaction of *T. cruzi* CL Brener and Dm28c/C2C12 and Caco-2 cells upon 2 and 24 h of infection. Our data showed that at two hours there is a strong cellular response mediated by EVs, both in the number, variety and enrichment/targeting of proteins found in LEVs for diverse functions. Qualitative and quantitative analysis showed that proteins exported in LEVs of C2C12 and Caco-2 have different patterns. We found a predominance of host proteins at early infection. The parasite-host cell interaction induces a switch in the functionality of proteins carried by LEVs and a heterogeneous response depending on the tissues analyzed. Protein–protein interaction analysis showed that cytoplasmic and mitochondrial homologues of the same parasite protein, tryparedoxin peroxidase, were differentially packaged in LEVs, also impacting the interacting molecule of this protein in the host. These data provide new evidence that the interaction with *T. cruzi* leads to a rapid tissue response through the release of LEVs, reflecting the enrichment of some proteins that could modulate the infection environment.

## Introduction

*Trypanosoma cruzi* is the protozoan that causes Chagas disease (CD)^1^, a parasitosis considered endemic in 21 countries in Latin America, with more than 6 million infected people^2^. *T. cruzi* has a very complex heteroxenous biological cycle, involving insect vectors of the Reduviidae family and mammalian hosts. Several aspects make the study of this pathogen more complex: the peculiar biology of the parasite and the transformations it undergoes, including multiple evolutionary stages; its adaptive capacity in different environments; the presence of distinct phenotypes between different strains and the pathophysiology of the disease, which involves an acute phase followed by persistence of the pathogen for many years in the host, that can develop a chronic phase of the disease.

Even after more than 100 years of its discovery, it is still not understood which factors determine the clinical evolution of CD. Host factors (such as the immune system) and parasite factors (such as differences between strains and infection routes) participate in the pathophysiology of the disease which generally involves myocardial and intestinal complications^3^. Recently, a series of works indicate that the gastrointestinal tract represents an important reservoir for *T. cruzi* in the chronic phase^4–8^. However, still little is known about the factors that influence the persistence and choice of the parasite by certain host tissues.

In this context, extracellular vesicles (EVs) may be acting as communicators between the parasite and host cells, facilitating invasion and modulating the immune system^9–16^. EVs are a diverse group of nanovesicles surrounded by a phospholipid bilayer constitutively released by either any prokaryotic or eukaryotic cell. The term EVs comprehends mainly two types of vesicles: exosomes, originated in multivesicular bodies of endocytic origin (ranging in diameter from 30 to 150 nm) and microvesicles (MVs), shed directly from the plasma membrane (100–1000 nm size). Due to the overlapping of physical characteristics (such as size) between MVs and exosomes and the difficulty of separating just one subpopulation with purity, the International Society of Extracellular Vesicles recommends the use of technical-operational terms, such as “Large Extracellular Vesicles” (LEVs, which correspond mainly to MVs) and “Small Extracellular Vesicles” (SEVs, mainly exosomes)^17^. Their packed and released cargos are very diverse and include lipids, proteins and different populations of nucleic acids and diverse biomolecules^18^. During contact between *T. cruzi* and host cells, there is an increase in the release of EVs, which constitute a mixture of parasite and host particles, including fusion between EVs^19^.

**Figure 1:**
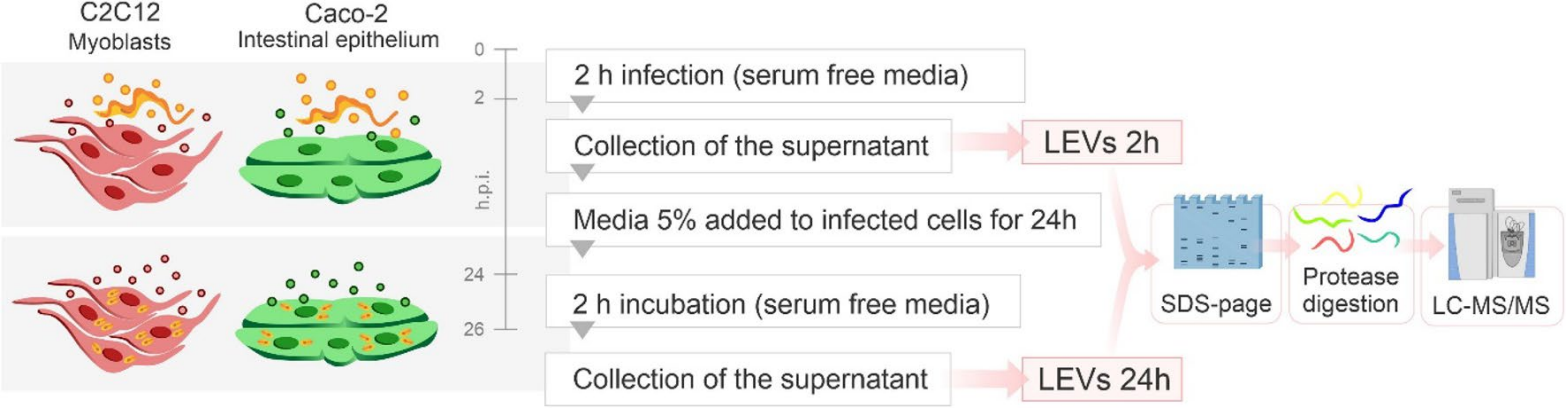
Workflow of the experimental design to obtain 2 and 24 h post infection (h.p.i.) LEVs. Myoblasts (C2C12) and intestinal epithelium (Caco-2) cells were seeded and infected with *T. cruzi* TCTs belonging to two distinct strains (CL Brener, DTU Tc VI and Dm28c DTU Tc I). LEVs were collected in the first 2 h and after 24 h of infection. LEVs derived from isolated C2C12, Caco-2 and *T. cruzi* TCTs were used as controls. LEVs were lysed, their proteins were separated and digested in-gel with trypsin and the peptides were analyzed by LC–MS/MS for protein identification and quantification.

In this study, we used two cell lines to simulate the environments found by the parasite in the host: mouse myoblast cell line C2C12, and human intestinal epithelial cell line Caco-2-. We isolated large EVs (LEVs) derived from C2C12 and Caco-2 cells after 2 h and 24 h of infection. For this, *T. cruzi* culture-derived trypomastigotes (TCTs) belonging to two strains with distinct genetic and phenotypic characteristics (CL Brener, belonging to DTU Tc VI and Dm28c belonging to DTU Tc I)^20–22^. Figure 1 summarize the general strategy of the study. Number, variety and functionality of proteins identified in LEVs of C2C12 and Caco-2 indicate different patterns of proteins exported in LEVs during the infection. We found a predominance of host proteins at early infection. The interaction of the parasite with the host cell induces a switch in the functionality of proteins carried by LEVs which also differs between myoblasts and epithelial intestinal cells. Furthermore, protein–protein interaction analysis indicates that LEVs carry key proteins for host–pathogen interaction, which could participate in the pathogenesis of CD.

## Methods

### Cell culture

Cell lines Vero (epithelial—monkey), C2C12 (myoblast—mouse), Caco-2 (intestinal epithelium—human) were obtained from the London Metropolitan University cell bank gently offered by Dr. Jameel Inal (London Metropolitan University, UK) and maintained in RPMI medium supplemented with 10% fetal bovine serum (FBS), at 37 °C with 5% CO_2_^23^. Tissue-derived trypomastigotes (TCT) from CL Brener (Tc VI) and Dm28c (Tc I) *T. cruzi* strains were obtained from the supernatant of Vero cells after 4–5 days of infection of the cells with TCTs (maintained with daily changes of RPMI 5% FBS medium)^24^.

### Induction and purification of LEVs

LEVs were isolated from the supernatant of the interaction of C2C12 or Caco-2 cells with *T. cruzi* TCTs in a 5:1 ratio (parasites:cell) in RPMI medium in the absence of FBS. Cells were plated in 6-well plates (5 × 10^5^ cells) 16–24 h before interaction. TCTs were washed in medium without FBS and added (2.5 × 10^6^ TCTs) to interact with cells for 2 h (37 °C, 5% CO_2_) in the absence of FBS. After 2 h incubation, the supernatant was collected for LEV isolation (then called 2 h LEVs). After collection, the cells (control or that were in contact with the parasites) received medium (5% FBS) and were incubated for another 24 h. After 24 h, the medium was replaced by RPMI without FBS and the supernatant was collected 2 h after changing the medium (called 24 h LEVs). LEVs derived from isolated C2C12, Caco-2 and *T. cruzi* TCTs were used as controls. Samples were obtained in duplicates. The collected supernatants were subjected to differential centrifugation for the isolation of LEVs. First, the supernatant was centrifuged at 500×*g* for 5 min. The supernatant was transferred to a new tube and centrifuged at 4000×*g* for 30 min to remove cellular debris. Them, LEVs were obtained by 11,000×*g*/120 min centrifugation. The pellets containing the LEVs were resuspended in PBS. Protein concentrations in EVs were determined using the Pierce-BCA protein assay kit (Thermo Scientific, USA) as described by the manufacturer.

### Protein preparation and analysis by mass spectrometry

Five micrograms of LEV proteins from duplicates of each condition were separated by 12% SDS-PAGE, stained with Coomassie Blue R-250, and submitted for in-gel protein digestion, as described elsewhere^25^ (Supplementary Fig. 1). Briefly, the whole gel lane for each sample was cut into approximately 1 mm^3^ cubes and destained (25 mM NH4HCO3 in 50% ethanol). Proteins were reduced (10 mM DTT in 100 mM NH4HCO3), alkylated (55 mM iodoacetamide in 100 mM NH4HCO3) and digested *in gel* with trypsin (sequencing grade modified, Promega) at a concentration of 12.5 ng/uL for 16 h at 37 °C. Peptides were extracted from the gel^26^ and desalted using C_18_-StageTips^24^ prior to analysis. Tryptic peptides were then separated by online reversed phase nanoscale capillary liquid chromatography (nanoLC) and analyzed by electrospray mass spectrometry in tandem (ESI–MS/MS) in a nanoLC-1D plus and autosampler as-2 (Eksigent) coupled to an LTQ Orbitrap XL ETD (Thermo Fisher Scientific). The peptide mixtures were separated in a packed emitter column with 15 cm length, 75 µm inner diameter, 3 µm C_18_ particles. Separation was carried out with a flow rate of 250 nL/min during 60 min using as mobile phases 0.1% acid formic, 5% DMSO in LC–MS water (phase A) and 0.1% formic acid, 5% DMSO in acetonitrile (phase B), with a linear gradient from 5 to 40% acetonitrile. Peptides were ionized by nanoelectrospray (2.7 kV) and injected into the mass spectrometer. Mass spectra were obtained by data-dependent acquisition in the orbitrap within a m/z window ranging from 300 to 1800, resolution of 60,000 at 400 m/z and AGC target of 10^6^. The “lock mass” option was enabled at 401.922718 m/z to improve mass accuracy. The MS/MS was carried out in the linear ion trap, where the 10 most intense precursor ions from each full scan were isolated at an AGC target of 3 × 10^4^ and fragmented by CID. The precursor ions were dynamically excluded for 90 s.

### Data analysis

For protein identification and quantification, raw data from the LC–MS/MS runs was analyzed in the MaxQuant platform^27^, version 2.2.0.0, using a reversed decoy databases of *T. cruzi* CL Brener (19,242 sequences), *T. cruzi* Dm28c (15,319 sequences), *Homo sapiens* (81,791 sequences), and *Mus musculus* (55,260 sequences), downloaded on march 22, 2023, from TritrypDB^28^ and UniprotDB^29^. The search parameters were MS tolerance of 20 ppm (Orbitrap), MS/MS tolerance of 0.5 Da (Iontrap), minimum peptide length of seven amino acids and trypsin with specific cleavage, allowing for two missed cleavages. Carbamidomethylation of cysteines was set as a fixed modification and methionine oxidation and protein N-terminal acetylation as variable modifications. The match between runs algorithm was used. A false discovery rate (FDR) of 1% was applied to the PSM and protein levels. After the removal of potential contaminants, reverse and ‘only identified by site’ hits, proteins identified in the two replicates of each condition were considered on further analyses. The hierarchical clustering was performed using the Euclidean distance in Perseus software v. 2.0.9^30^, the intensity (XIC values) of the proteins were transformed to log2 and the missing values were replaced by the normal distribution values of the data set (width: 0.3; down shift: 1.8). In quantitative analysis we use MaxQuant to calculate iBAQ value of proteins. The iBAQ value is obtained by dividing protein intensities by the number of theoretically observable tryptic peptides^31^. Proteins with iBAQ values calculated in at least 2 replicates per condition were Log2 transformed and considered in the t-test analysis with a permutation-based FDR calculation using FDR 0.05 and S0 0.1. The functional analysis of proteins found in LEVs was performed by gene ontology (G.O.) enrichment analysis (https://geneontology.org/)^32^. The subcellular localization analysis of LEV proteins was carried out using the website DeepLoc (https://services.healthtech.dtu.dk/services/DeepLoc-2.0)^33^ and cellular localizations with a presence greater than 3% in all EVs are presented in the graphs. The Venn diagrams were created using the BioVenn tool^34^. The protein–protein interactions were visualized using the Search Tool for the Retrieval of Interacting Genes/Proteins (STRING)^35^ in Cytoscape stringApp 2.0^36^ with high-confidence score cutoff of 0.90 and organic layout to group and locate the nodes.

## Results

### Protein patterns in LEVs released from myoblast and intestinal epithelium cells during *T. cruzi* infection highlight host dominance at early infection

Based on recent findings that the gastrointestinal tract represents an important reservoir for *T. cruzi* in the chronic phase and that the muscle tissues are particularly compromised during the pathogenesis of CD^4–8^, we hypothesized that the interaction of the parasite with the intestinal and muscular tissues, could result in different patterns of extracellular vesicle release. In order to investigate the hypothese, we used mass spectrometry-based proteomics to analyze the protein content of LEVs released in vitro by intestinal and muscular cell lines upon 2 h of interaction and 24 h of infection with *T. cruzi* TCTs (Fig. 1). The size distribution of LEVs in each sample shown that the method was able to mostly isolate LEVs and identify their proteins (Supplementary Fig. 2A,B, respectively).

**Figure 2:**
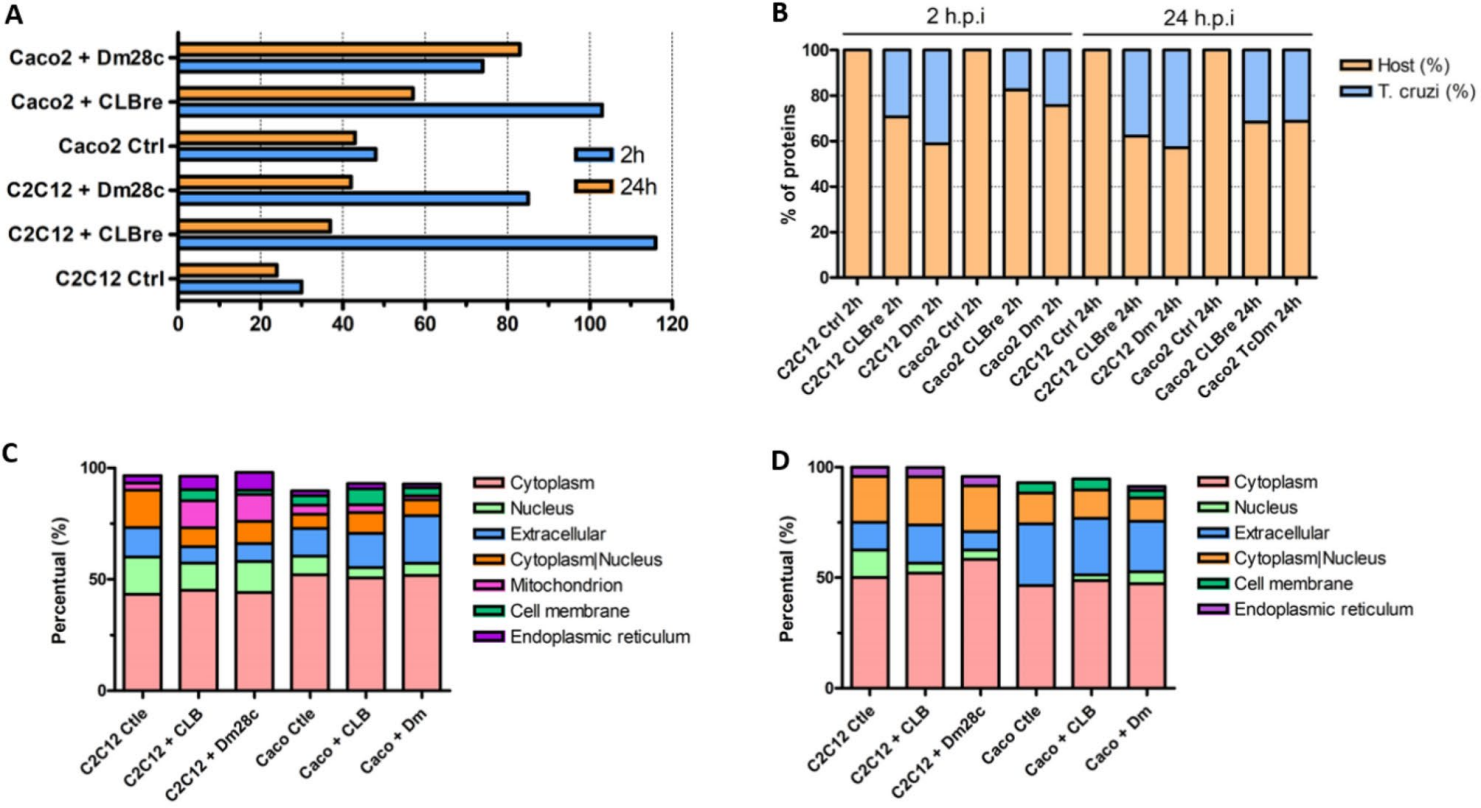
(**A**) Number of proteins identified in the 2 h and 24 h LEVs of myoblasts (C2C12) and epithelial cells (Caco-2) cells with or without contact with *T. cruzi*. (**B**) Proportion of host (mouse or human) and *T. cruzi* proteins identified in LEVs. (**C**) Subcellular localization of host proteins present in LEVs 2 h. (**D**) Subcellular localization of host proteins present in LEVs 24 h.

We identified a greater variety of proteins in LEVs released after the first two hours of interaction with the parasite, compared to those from 24 h post infection (h.p.i.), except for intestinal epithelium (Caco-2) interacting with *T. cruzi* Dm28c (Fig. 2A). When looking into proteins of host and parasite origin, it is possible to notice that contact with the parasite for 2 h induces a greater secretion of host proteins in LEVs, which equals after 24 h of infection (Fig. 2B). In addition, a higher proportion of host proteins were identified in LEVs derived from the interaction of the parasite with Caco-2 (82.5% and 75.7% for CL Brener and Dm28c, respectively), when compared to myoblasts (C2C12) (70.7% and 58.8%, for CL Brener and Dm28c, respectively) cells. Detailed results are available in Supplementary Table 1.

To understand whether the profile of host proteins present in LEVs released in the different contexts studied would also differ, we analyzed the subcellular localization of the protein sets found in each sample (Supplementary Table 2). Cytoplasmic proteins are the majority (> 40%) and membrane and endoplasmic reticulum proteins had a lower proportion in all LEVs analyzed (less than 8%), both at 2 h.p.i. (Fig. 2C) and 24 h.p.i. (Fig. 2D). Despite the general cytoplasmic majority, a predominance of different sublocations in different samples was observed. For example, in LEVs derived from C2C12 after the first 2 h.p.i. (with or without contact with parasites), an expressive number (> 10%) of nuclear proteins is seen. On the other hand, extracellular proteins appeared in greater proportion (> 20%) in Caco-2 LEVs, mainly after 24 h.p.i. At the moment, we do not know if these proteins could have been “pelleted” together with the EVs (as contaminants) or if they represent a phenomenon of extracellular protein export in the EVs, the biological value of this data still needs to be explored and validated. Also, mitochondrial proteins appeared only in EVs derived from the first two hours of infection in all LEVs.

### Qualitative and quantitative analysis of proteins identified in LEVs indicate a different export profile in host cells under interaction with the *T. cruzi*

**Figure 3:**
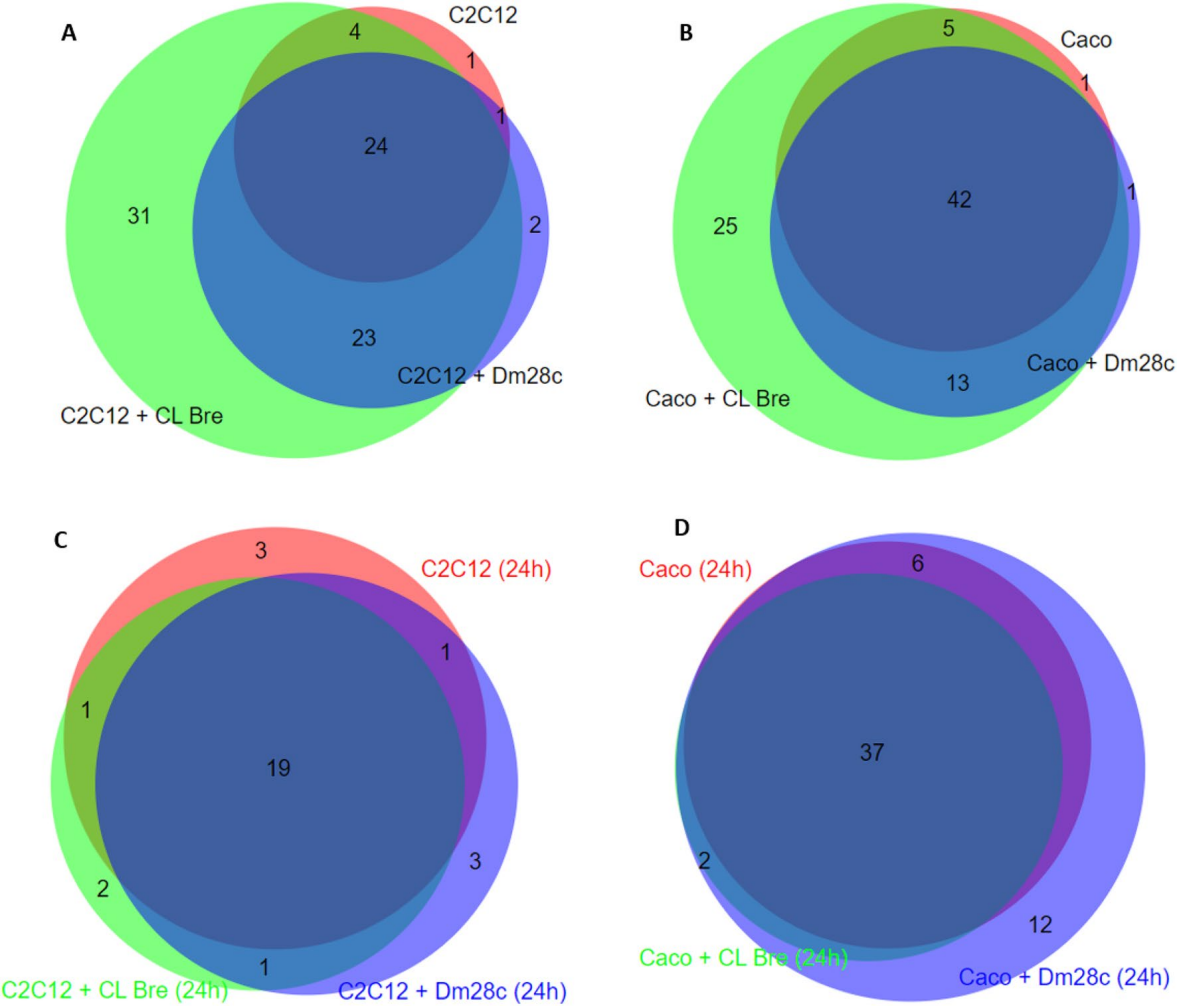
Venn diagrams of proteins present in LEVs. (**A**) 2 h LEVs of myoblasts (C2C12). (**B**) 2 h LEVs of epithelial cells (Caco-2). (**C**) 24 h LEVs of myoblasts (C2C12). (**D**) 24 h LEVs of epithelial cells (Caco-2).

The qualitative analysis of the proteins identified in the LEVs showed that, among the control LEVs (cells without contact with parasites for 2 and 24 h), four proteins are common to all (annexin A2, fructose biphosphate aldolase, plakoglobin and tubulin alpha 1-B) (Supplementary Fig. 3), two of which (annexin and tubulin) are commonly found in EVs^37^. On the other hand, the overlap of identified proteins is greater between the control samples of each cell line (32 common proteins in Caco-2 and 18 in C2C12), demonstrating the heterogeneous profile of LEV proteins from different cell lines. Despite the high number of proteins shared between 2 h LEVs in control and infected cells (27.9% for C2C12 and 48.3% for Caco-2), we identified 23 (26.7%) and 13 (14.9%) proteins found exclusively in interaction of both strains with myoblasts and epithelial cells, respectively (Fig. 3A,B and Table 1). Among them, we found proteins involved in metabolism, cytoskeleton, immunoglobulins, and cellular transport. In 24 h LEVs, we identified a smaller number of proteins exclusive from the interaction (heat shock protein HSP 90-beta for myoblast and plakophilin-1 and histone H4 for epithelial cells (Fig. 3C,D).

**Table 1:** Host proteins exclusively identified in 2 h LEVs upon *T. cruzi* infection.

2 h LEVs myoblasts + *T. cruzi*	2 h LEVs epithelial cells + *T. cruzi*
1. Sodium/potassium-transporting 2. ATPase subunit alpha 3. Collagen, type VI, alpha 5 4. Eukaryotic initiation factor 4A-I 5. ATP synthase subunit alpha 6. Heat shock protein 7. HSP 90-beta 8. Superoxide dismutase 9. Calreticulin 10. 40S ribosomal protein S18 11. Tubulin beta-5 chain 12. rRNA/tRNA 2-O-methyltransferase fibrillarin-like protein 1 13. Histone H1.2 14. Heterogeneous nuclear ribonucleoprotein U 15. Phosphate carrier protein, mitochondrial 16. Vimentin 60 kDa heat shock protein, mitochondrial 17. Clathrin heavy chain 1 18. ATP synthase subunit beta, mitochondrial 19. Protein disulfide-isomerase 20. Endoplasmic reticulum chaperone 21. BiP 40S ribosomal protein S4, X isoform 22. Endoplasmin ADP/ATP translocase 2 23. Myosin-9	1. Tyrosine 3-monooxygenase/tryptophan 5-monooxygenase activation protein zeta (Fragment) 2. Semenogelin-1 3. Zinc-alpha-2-glycoprotein 4. Extracellular glycoprotein lacritin 5. Small proline-rich protein 2A 6. Cystatin-A 7. Prolactin-inducible protein 8. Myosin heavy chain 9 9. Immunoglobulin heavy constant gamma 1 (Fragment) 10. Clathrin heavy chain 1 11. Peptidyl-prolyl cis–trans isomerase A 12. Immunoglobulin heavy constant alpha 2 (Fragment) 13. Plakophilin-1

**Figure 4:**
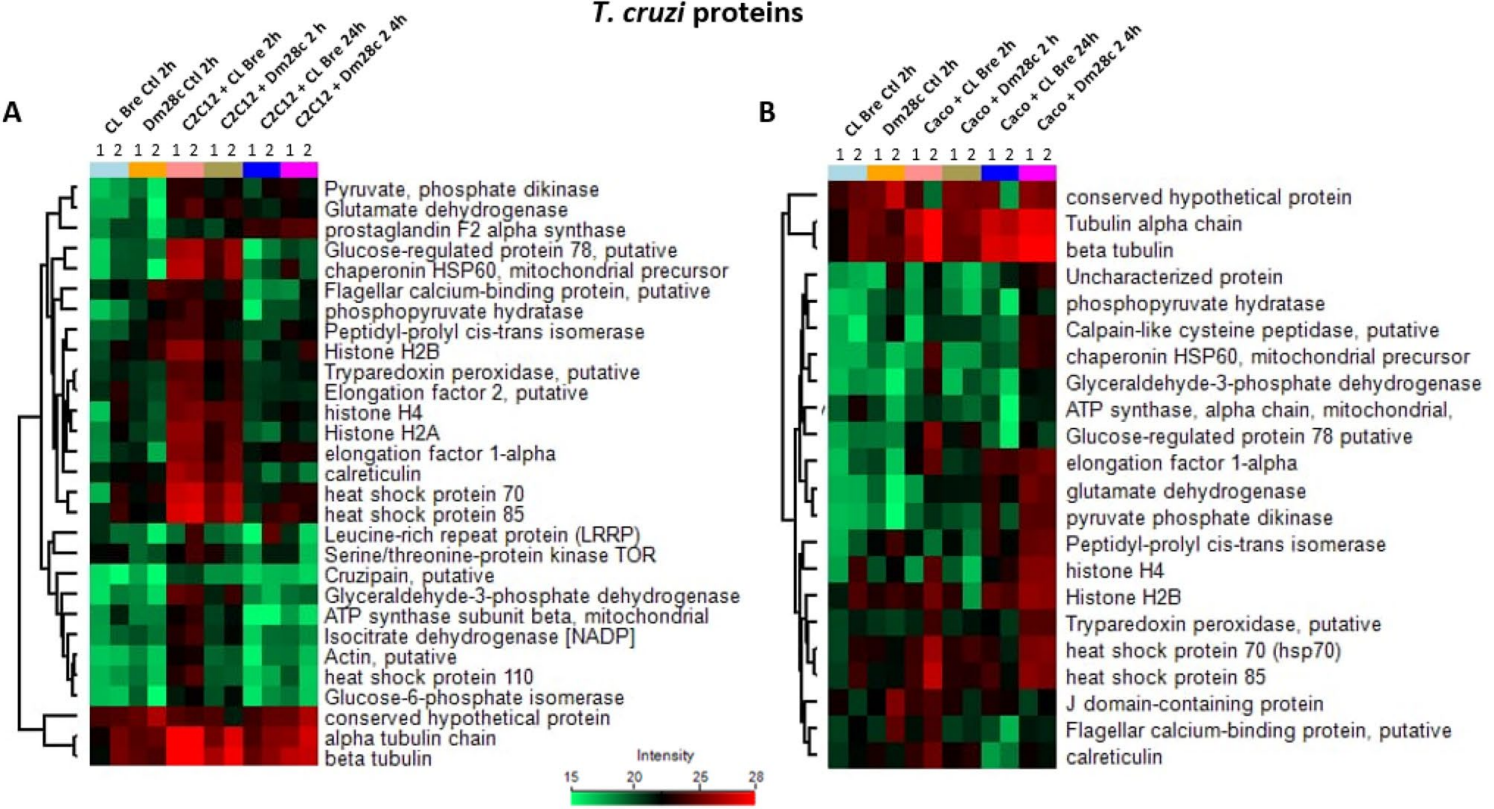
Hierarchical clustering of *T. cruzi* LEVs proteins. Heatmaps showing the expression level of LEV proteins from *T. cruzi* strains CL Brener and Dm28c isolated and during interaction for 2 h and 24 h with myoblasts (C2C12) (**A**) and epithelial cells (Caco-2) (**B**). Each condition is represented by a color and numbered according to the replicate. CL Bre Ctl and Dm28c Ctl correspond to LEV proteins coming from the isolated parasite (without contact with host cells) for 2 h; the other samples refer to the LEVs proteins coming from the contact of each strain with the host cell for 2 h or 24 h.

**Figure 5:**
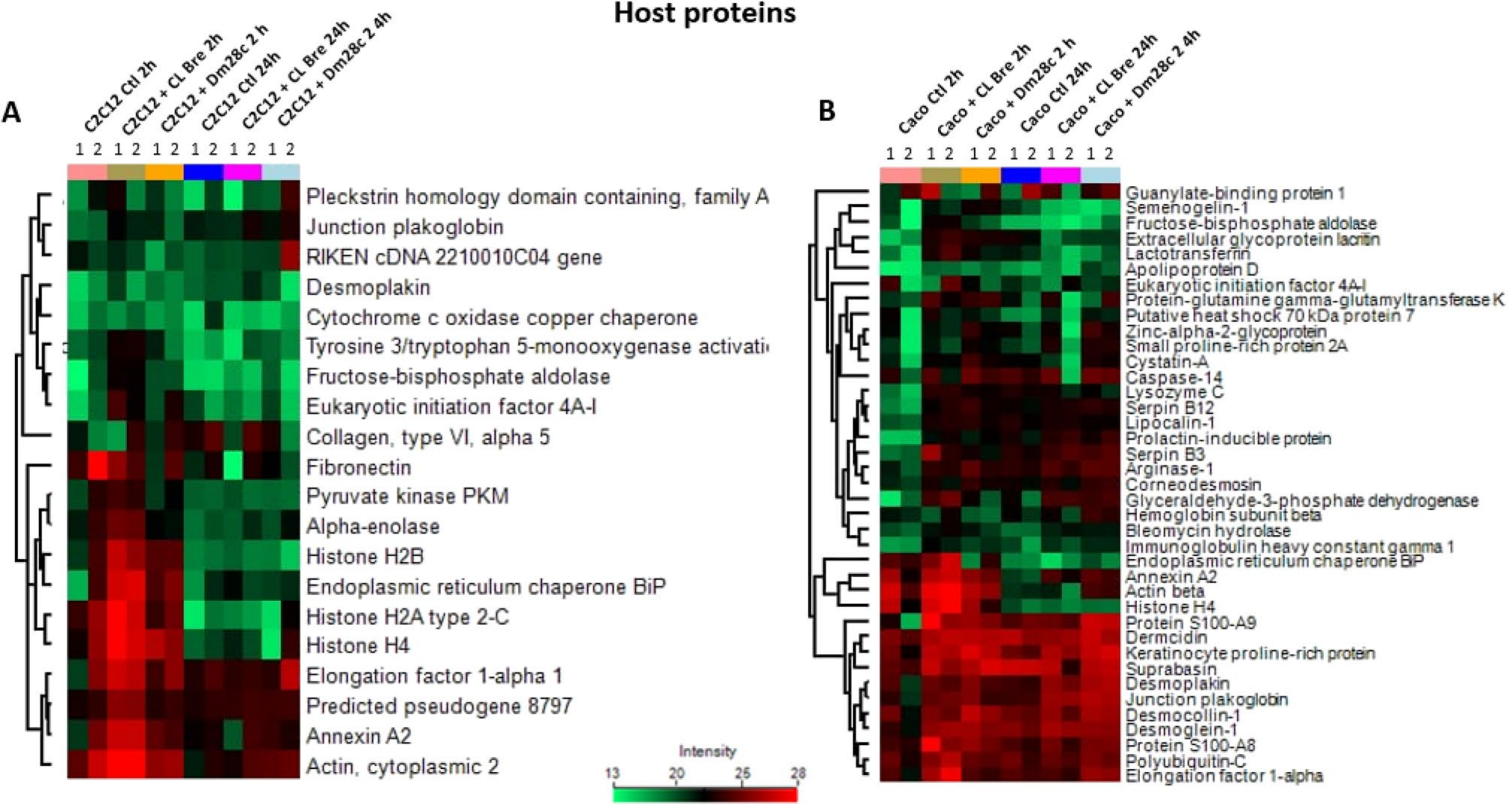
Hierarchical clustering of host cell LEV proteins. Heatmaps showing the expression level of LEV proteins from myoblasts (C2C12) (**A**) and epithelial cells (Caco-2) (**B**) isolated and under interaction for 2 h and 24 h with *T. cruzi* CL Brener and Dm28c strains. Each condition is represented by a color and numbered according to the replicate. C2C12 Ctl and Caco-2 Ctl correspond to LEV proteins coming from the isolated host cells (without contact with *T. cruzi*) for 2 h; the other samples refer to the LEV proteins coming from the contact of host cell with each *T. cruzi* strain with the for 2 h or 24 h.

The expression profile analysis of the proteins allowed the identification of different clusters in the analyzed conditions (Figs. 4 and 5). For *T. cruzi* proteins (Fig. 4), it is possible to see that tubulin (alpha tubulin chain and beta tubulin) was consistently identified. Furthermore, a conserved hypothetical protein (TriTrypDB ID: C4B63_40g107), annotated as basal body protein and orthologous protein in other *T. cruzi* strains, was also found in all EVs. Proteins such as tryparedoxin peroxidase, elongation factor 1-alpha and 2, histones H4, H2A and H2B, calreticulin and heat shock protein 70 and 85 increased in LEVs of myoblasts (C2C12) with *T. cruzi* in the first 2 h of infection, but decreased after 24 h (Fig. 4A). For epithelial cells (Caco-2), heat shock protein 70 and 85 appear with a constant profile in all samples (Fig. 4B).

Regarding host cell proteins identified in the LEVs (Fig. 5), it is possible to notice that proteins such as actin, annexin A2, elongation factor 1-alpha and fibronectin remain abundant in all LEVs coming from myoblasts (C2C12) (with or without contact with *T. cruzi*) (Fig. 5A). It is also possible to note that certain proteins are abundant in 2 h LEVs and have a low detection in LEVs coming from cells 24 h.p.i. In epithelial (Caco-2) (Fig. 5B), a high concentration of proteins such as suprabasin, keratinocyte protein rich in proline, dermcidin, desmoplakin, plakoglobin, desmocollin, desmoglein, polyubiquitin-C and elongation factor 1-alpha was seen in all samples. Interestingly, some of these proteins are important in cell adhesion processes.

**Figure 6:**
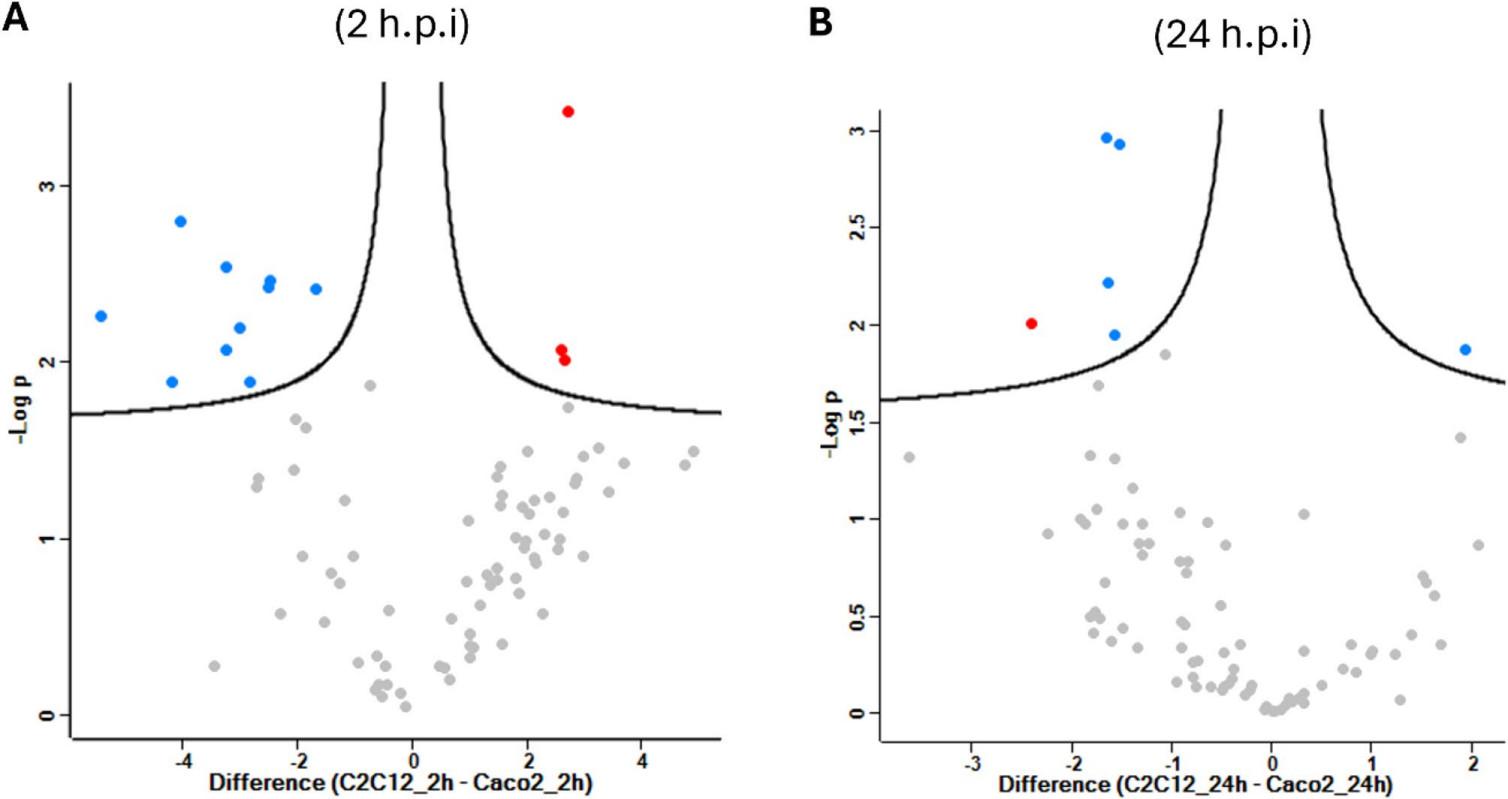
Differentially abundant proteins in LEVs. Volcano plots representing statistically significant proteins identified in LEVs of interaction between *T. cruzi* with C2C12 and Caco-2 during 2 h.p.i (**A**) and 24 h.p.i. (**B**). Statistically significant proteins are in red (host proteins) and blue (*T. cruzi* proteins).

In order to identify differentially abundant proteins between conditions involving the parasite and host cells, we compared the iBAQ values of proteins between *T. cruzi* and different host cells for in 2 h and 24 h h conditions (Supplementary Table 6). The analysis reveals 13 differentially abundant proteins in parasite interaction with C2C12 and Caco-2 during 2 h (Fig. 6A), and 6 proteins in 24 h post infection (Fig. 6B). Differentially abundant proteins related to adhesion and immune response were identified in the vesicles of host cells while from parasite were identified proteins related to carbohydrate and amino acid metabolism, and this response was more intense in the 2 h.p.i. and Caco-2 cell (Table 2).

**Table 2:** Differentially abundant proteins identified in the LEVs of C2C12 and Caco-2 cells interacting with *T. cruzi.*

Protein name	IDs	Cell/strain	-LOG(P-value)	Difference
Tc-C2C12 × Tc-Caco-2 (2 h.p.i.)				
Immunoglobulin heavy constant gamma 1 (Fragment)	A0A0A0MS07	Caco-2	2.424	− 2.513
Pyruvate phosphate dikinase	C4B63_12g221	Tc Dm28c	2.070	2.594
Peptidyl-prolyl cis-trans isomerase	Q4E4L9; C4B63_19g183	Tc CL Brener; Tc Dm28c	3.427	2.720
Phosphopyruvate hydratase	Q4DZ98; C4B63_47g72	Tc CL Brener; Tc Dm28c	2.011	2.669
Arginase-1	P05089	Caco-2	2.539	− 3.249
Protein S100-A8	P05109	Caco-2	2.194	− 3.002
Protein S100-A9	P06702	Caco-2	2.799	− 4.043
Lysozyme C	P61626; P17897	Caco-2; C2C12	1.888	− 2.823
Putative lipocalin 1-like protein 1	Q5VSP4	Caco-2	1.885	− 4.201
Dermcidin	P81605	Caco-2	2.417	− 1.685
Desmocollin-1	Q08554	Caco-2	2.459	− 2.474
Keratinocyte proline-rich protein	Q5T749	Caco-2	2.070	− 3.230
Extracellular glycoprotein lacritin	Q9GZZ8	Caco-2	2.264	− 5.416
Tc-C2C12 × Tc-Caco-2 (24 h.p.i.)				
Corneodesmosin	Q2L6G8	Caco-2	2.216	− 1.637
Protein S100-A9	P06702	Caco-2	1.953	− 1.570
Junction plakoglobin	Q02257; P14923	C2C12; Caco-2	2.935	− 1.517
Desmoplakin	P15924; E9Q557	Caco-2; C2C12	2.968	− 1.654
Histone H2A (Fragment)	Q4CYH9	Tc CL Brener	2.004	− 2.405
Extracellular glycoprotein lacritin	Q9GZZ8	Caco-2	1.873	1.934

All together, our analysis of LEV proteins indicate that 24 h after interaction with *T. cruzi*, there is a lower cellular response through the release of EVs.

### The parasite-host interaction induces a switch in the functionality of proteins carried by LEVs in early infection

**Figure 7:**
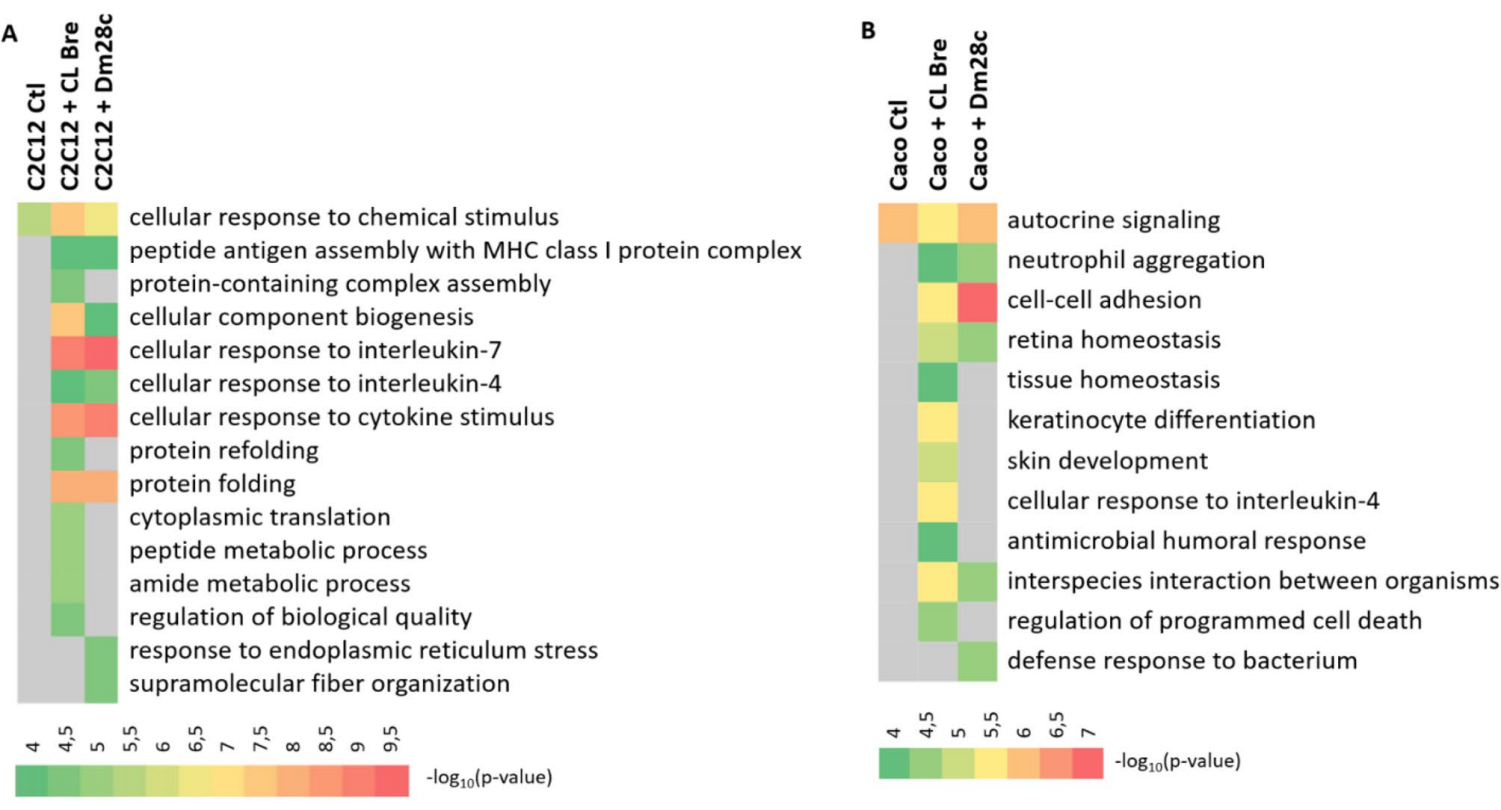
Biological processes enriched in C2C12 (**A**) and Caco-2 (**B**) LEV proteins during 2 h.p.i. with each *T. cruzi* strain. Colors indicate the value of -log_10_(*p*-value). Terms indicated in gray were not significantly enriched in the condition.

In order to understand the functions of the host proteins carried by their LEVs, we analyzed the biological processes enriched in which these proteins would be involved. All enriched categories (*p*-value < 0.05) are shown in Supplementary Table 3 and some main processes are shown in Fig. 7. Interestingly, LEVs 2 h from control cells (which had no contact with *T. cruzi*) were enriched for one GO term only: cellular response to chemical stimulation for myoblasts (C2C12) (Fig. 7A) and autocrine signaling for epithelial cells (Caco-2) (Fig. 7B). On the other hand, when cells interact with *T. cruzi*, more GO terms appear enriched, showing that the composition of EVs is affected by exposure to the pathogen. Biological processes of cell response, such as response to cytokines/interleukins, protein folding, peptide antigen assembly with MHC I, neutrophil aggregation, regulation of cell death, and biological processes involved in inter-species interaction and cell adhesion appear over-represented in 2 h LEVs from cells in contact with *T. cruzi.*

### LEVs carry key proteins for parasite-host interaction

**Figure 8:**
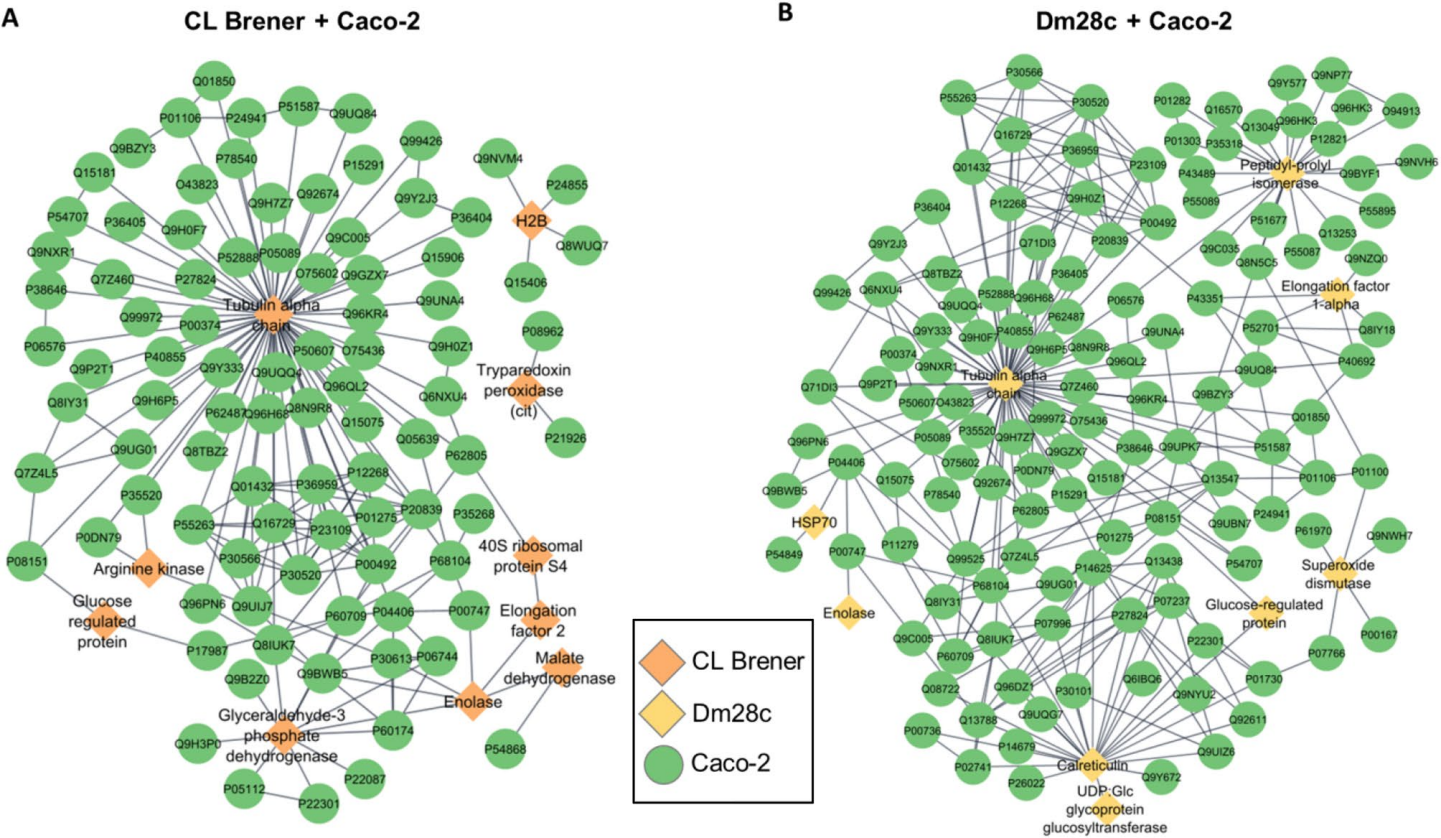
Interactome of LEV proteins of *T. cruzi* CL Brener (**A**) and Dm28c (**B**) strains with epithelial cells (Caco-2). Circles: Caco-2 proteins, diamonds: *T. cruzi* proteins.

**Figure 9:**
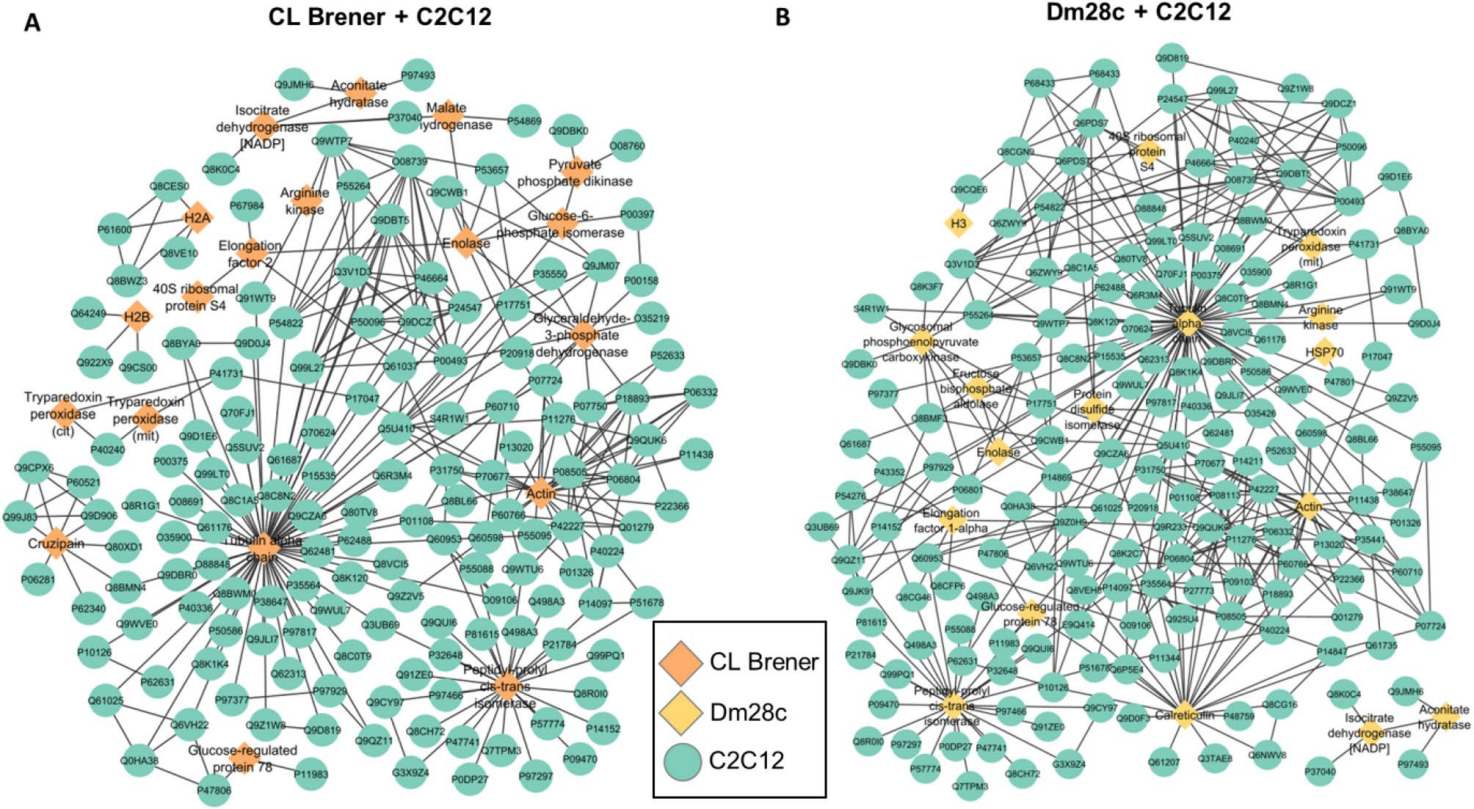
Interactome of LEV proteins of *T. cruzi* CL Brener (**A**) and Dm28c (**B**) strains with myoblast (C2C12). Circles: C2C12 proteins, diamonds: *T. cruzi* proteins.

Taking into consideration that EVs carry parasitic molecules, we screened in silico for potential protein–protein interactions (PPI) between pathogen LEV proteins and host cells. We identified 438 potential interactions between *T. cruzi* (CL Brener 178; Dm28c 260) and epithelial cells (Fig. 8), and 689 interactions between *T. cruzi* (CL Brener 312; Dm28c 377) and myoblast cells (Fig. 9) (Supplementary Table 4). These interactions are carried out by 10 proteins from the CL Brener strain (Fig. 8A) and 9 from the Dm28c strain (Fig. 8B), interacting with 92 and 131 proteins from the epithelial cells, respectively. In the parasite interaction with myoblast cells, 19 proteins from the CL Brener strain (Fig. 9A) and 17 from the Dm28c strain (Fig. 9B) were mapped, interacting with 152 and 168 proteins, respectively.

An important virulence factor mapped by PPI analysis was tryparedoxin peroxidase (TP), which is an important component in the antioxidant system of *T. cruzi* during host infection^38^. *T. cruzi* has a cytosolic TP and a mitochondrial TP^39^. Both TPs were identified, but in different parasite-host cell conditions. For example, in the CL Brener-epithelial cells interaction only cytosolic TP (Q4CVR5) was identified, while in the interaction of this same strain with myoblast, cytosolic and mitochondrial TP (Q4CX87) were identified. In the Dm28c-epithelial cells interaction, neither of the two TPs was identified, whereas in the interaction of this strain with myoblasts, the mitochondrial TP was identified.

Different interaction partners were mapped according to the type of host cell. For example, in the interaction with epithelial cells (Caco-2), the cytosolic TP (Q4CVR5) interacts with Lysosomal-associated membrane protein and with cell growth-inhibiting gene 2 protein (Fig. 8). In the interaction with myoblast (C2C12), the mitochondrial TP (Q4CX87) interacts with the CD63 and CD9 antigens (Fig. 9) opening the idea that the dynamic of interaction between pathogens and host cells could be varied and different trough LEVs in each tissue.

## Discussion

Chagas disease represents a spectrum of pathogenesis, varying both in its severity and in the organs affected. The fine balance between various host factors (such as the immune response) and parasitic factors determines the outcome of the infection and its pathogenesis. The present study was carried out to evaluate the extent to which the release of extracellular vesicles could help to elucidate the interaction of *T. cruzi* with different tissues. Our proteomics results showed that LEVs coming from myoblast (C2C12) and epithelial cells (Caco-2) cells have different components, and that the infection influences the composition of the LEVs. We have seen a difference in the ratio of host and parasite proteins between two *T. cruzi* strains at 2 h and 24 h after infection. Our data showed that during the first two hours of contact with *T. cruzi*, there is a strong cellular response mediated by EVs, both in the number of proteins found in LEVs and in the enrichment/targeting of proteins for diverse functions. The work of Ramirez et al.^19^ shows that the interaction of *T. cruzi* metacyclics and TCTs with THP-1 macrophages (for 1 h) contribute proteins to the formation of EVs, with 25% (metacyclics) and 12% (TCTs) of the proteins found in the EVs under these conditions belonging to *T. cruzi*, while only 5% of the proteins are parasitic in the interaction with epimastigotes. In our results, the percentage of parasitic proteins found in LEVs varies from approximately 20–40%, a number that may be higher due to the longer interaction time (2 h). Our data show 30–50 proteins found in control EVs (C2C12 and Caco-2), which is similar to that reported by Ramirez et al. 2017 (54 THP-1 proteins in the absence of parasites). In the same way as in our results, Ramirez et al. 2017 reported that the number of total proteins increases upon interaction with *T. cruzi* (from 54 proteins in the control to 91 and 110 when interacting with metacyclics and TCTs, respectively). Interestingly, the authors show that parasite and host EVs fuse^19^, which could hypothetically result in better uptake of these particles in certain tissues, depending on the components they carry.

Bautista-López et al.^40^ characterized EVs secreted from Vero cells infected with *T. cruzi* and showed that only 10% of the total proteins were of parasitic origin. The group of *T. cruzi* proteins most abundant in EVs were from the trans-sialidases family, which was also found in another study that evaluated EVs from isolated parasites^11^, however, these proteins were not very representative in our data. We identified three trans-sialidases (groups I, II and V) in the *T. cruzi*-myoblast interaction and only one (group I) in the *T. cruzi*-epithelial cells interaction. This difference is probably because these previous studies evaluated EVs coming from cells on the 4th day after infection (where the parasites have already replicated and differentiated into trypomastigotes, which express high levels of trans-sialidases)^41^, while our data were obtained within 24 h after infection, when the parasites are just beginning to replicate as amastigotes.

Cronenberger-Andrade et al.^15^ evaluates the secretion of EVs from macrophages (THP-1) infected by *T. cruzi.* A total of 123 proteins were found in EVs from uninfected THP1 and 89 were found in EVs from infected cells, out of a total of 154 proteins in both samples. Interestingly, *T. cruzi* proteins found in infected THP-1 EVs such as HSP60, tryparedoxin peroxidase, trans-sialidase Group II, flagellar calcium-binding protein were also found in our experiment (Table S1). Most of the proteins found in EVs are involved in binding, immunological and metabolic processes.

Parasite and host proteins identified in the present study were compared with proteins found in the works of Ramirez et al.^19^, Bautista-López et al.^40^ and Cronenberger-Andrade et al.^15^. Fifteen proteins were identified in common with those published by Ramirez et al. (2017) in 2 h LEVs and 7 proteins in 24 h LEVs (Supplementary Table 5). Among the functions observed in these proteins are binding, catalytic activity, structural molecule activity, and translation regulatory activity.

Although our results show that LEVs carry important parasitic and cellular components, it remains an open question whether they have the capacity to influence the parasite’s tropism for certain host tissues. A limitation of experimental work with *T. cruzi* is not being able to simulate the complex environment of chronic infection in vitro. Trying to simulate possible environments found by parasites, we used cell lines that would represent muscle and intestinal tissues to study their EVs. It was possible to evidence a considerable difference between LEVs derived from the myoblasts and epithelial cells lines. However, it is important to highlight that a pattern of strong cellular response to infection was seen and that LEVs released from these cells could carry information to adjacent tissues as a warning signal. An enrichment of adhesion-related proteins was noted in LEVs derived from epithelial cells (Caco-2) in contact with the parasite.

Our data also show the ability of LEVs to carry virulence factor from parasites. For example, one of the proteins detected in LEVs was cruzipain, a highly mannosylated cysteine protease that has been described as cleaving kininogen in host cells, creating short-lived kinins which bind to the bradykinin receptor to stimulate IP3-mediated Ca2 + release, with such a pathway being associated with parasite internalization^42^. Another important factor found in LEVs is calreticulin, a molecule that inhibits both the classical and lectin complement pathways, protecting the parasite from lysis^43^. These data open the way for new studies that investigate the role of EVs in modulating host responses during CD.

### Supplementary Information

Supplementary Legends.

Supplementary Table S1.

Supplementary Table S2.

Supplementary Table S3.

Supplementary Table S4.

Supplementary Table S5.

Supplementary Table S6.

Supplementary Information 8.

## Data Availability

The mass spectrometry proteomics data have been deposited to the ProteomeXchange Consortium via the PRIDE^44^. Partner repository with the dataset identifier PXD046906.
