# Supplementary material for: Trypanosoma cruzi interaction with host tissues modulate the composition of large extracellular vesicles
Source: Sci Rep. 2024 Feb 29;14:5000. doi: 10.1038/s41598-024-55302-3 (PMC10904747; doi:10.1038/s41598-024-55302-3)
Supplement: Supplementary file 1 — Supplementary Legends. [file 41598_2024_55302_MOESM1_ESM.docx]

Fig. 1. Workflow of the experimental design to obtain 2 and 24 hours post infection (h.p.i.) LEVs. Myoblasts (C2C12) and intestinal epithelium (Caco-2) cells were seeded and infected with *T. cruzi* TCTs belonging to two distinct strains (CL Brener, DTU Tc VI and Dm28c DTU Tc I). LEVs were collected in the first 2 hours and after 24 hours of infection. LEVs derived from isolated C2C12, Caco-2 and *T. cruzi* TCTs were used as controls. LEVs were lysed, their proteins were separated and digested in-gel with trypsin and the peptides were analyzed by LC-MS/MS for protein identification and quantification.

Fig. 2. A) Number of proteins identified in the 2 h and 24 h LEVs of myoblasts (C2C12) and intestinal epithelium (Caco-2) cells with or without contact with *T. cruzi*. B) Proportion of host (mouse or human) and *T. cruzi* proteins identified in LEVs. C) Subcellular location of host proteins present in LEVs 2 h. D) Subcellular location of host proteins present in LEVs 24 h.

Fig. 3. Venn diagrams of proteins present in LEVs. A) 2h LEVs of myoblasts (C2C12). B) 2h LEVs of intestinal epithelium (Caco-2). C) 24h LEVs of myoblasts (C2C12). D) 24h LEVs of intestinal epithelium (Caco-2).

Fig 4. Hierarchical clustering of *T. cruzi* LEVs proteins. Heatmaps showing the expression level of LEV proteins from *T. cruzi* strains CL Brener and Dm28c isolated and during interaction for 2 h and 24 h with myoblasts (C2C12) (A) and intestinal epithelium (Caco-2) (B) cells. Each condition is represented by a color and numbered according to the replicate. CL Bre Ctl and Dm28c Ctl correspond to LEV proteins coming from the isolated parasite (without contact with host cells) for 2 h; the other samples refer to the LEVs proteins coming from the contact of each strain with the host cell for 2 h or 24 h.

Fig 5. Hierarchical clustering of host cell LEV proteins. Heatmaps showing the expression level of LEV proteins from myoblasts (C2C12) (A) and intestinal epithelium (Caco-2) (B) cells isolated and under interaction for 2 h and 24 h with *T. cruzi* CL Brener and Dm28c strains. Each condition is represented by a color and numbered according to the replicate. C2C12 Ctl and Caco-2 Ctl correspond to LEV proteins coming from the isolated host cells (without contact with *T. cruzi*) for 2 h; the other samples refer to the LEV proteins coming from the contact of host cell with each T. cruzi strain with the for 2 h or 24 h.

Fig. 6. Differentially abundant proteins in LEVs. Volcano plots representing statistically significant proteins identified in LEVs of interaction between *T. cruzi* with C2C12 and Caco-2 during 2 h.p.i (A) and 24 h.p.i. (B). Statistically significant proteins are in red (host proteins) and blue (*T. cruzi* proteins).

Fig 7. Biological processes enriched in C2C12 (A) and Caco-2 (B) LEV proteins during 2 h.p.i. with each *T. cruzi* strain. Colors indicate the value of -log_10_(p-value). Terms indicated in gray were not significantly enriched in the condition.

Fig. 8. Interactome of LEV proteins of *T. cruzi* CL Brener (A) and Dm28c (B) strains with intestinal epithelium (Caco-2). Circles: Caco-2 proteins, diamonds: *T. cruzi* proteins.

Fig. 9. Interactome of LEV proteins of *T. cruzi* CL Brener (A) and Dm28c (B) strains with myoblast (C2C12). Circles: C2C12 proteins, diamonds: *T. cruzi* proteins.
