# Supplementary material for: Trypanosoma cruzi interaction with host tissues modulate the composition of large extracellular vesicles
Source: Sci Rep. 2024 Feb 29;14:5000. doi: 10.1038/s41598-024-55302-3 (PMC10904747; doi:10.1038/s41598-024-55302-3)
Supplement: Supplementary file 7 — Supplementary Table S6. [file 41598_2024_55302_MOESM7_ESM.pdf]

FIGURES

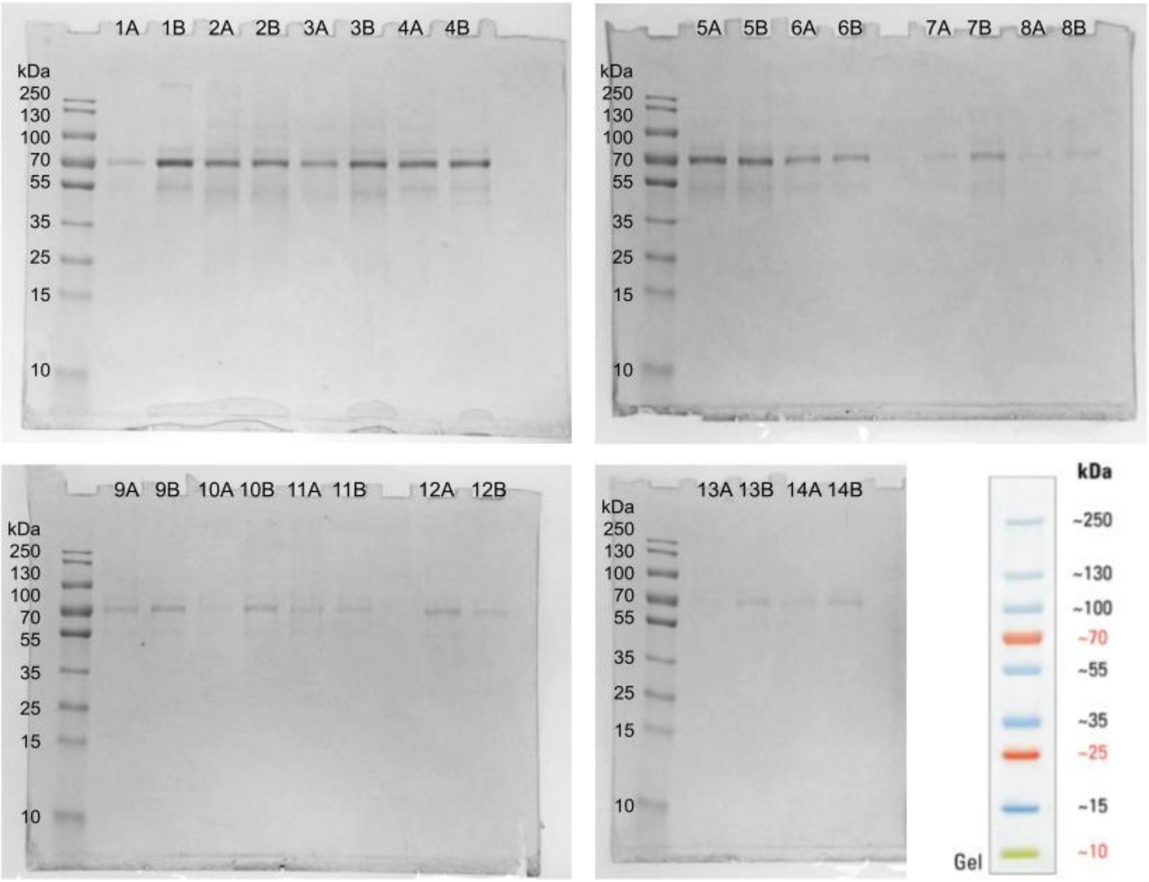

Supplementary Figure 1

Images of the SDS-page gels where five micrograms of LEV proteins from duplicates of each condition were applied. 12% SDS-PAGE gel was stained with Coomassie Blue R-250 and then subjected to in-gel protein digestion. LEVs: 1. EVs C2C12 Ctl (2h). 2. EVs C2C12 + CLBre (2h). 3. EVs C2C12 + Dm28c (2h). 4. EVs Caco-2 Ctl (2h). 5. EVs Caco-2 + CLBre (2h). 6. EVs Caco-2 + Dm28c (2h). 7. EVs CLBre (2h). 8. EVs Dm28c (2h). 9. EVs C2C12 Ctl (24h). 10. EVs C2C12 + CLBre (24h). 11. EVs C2C12 + Dm28c (24h). 12. EVs Caco-2 Ctl (24h). 13. EVs Caco-2 + CLBre (24h). 14. EVs Caco-2 + Dm28c (24h). A and B indicates two replicates.

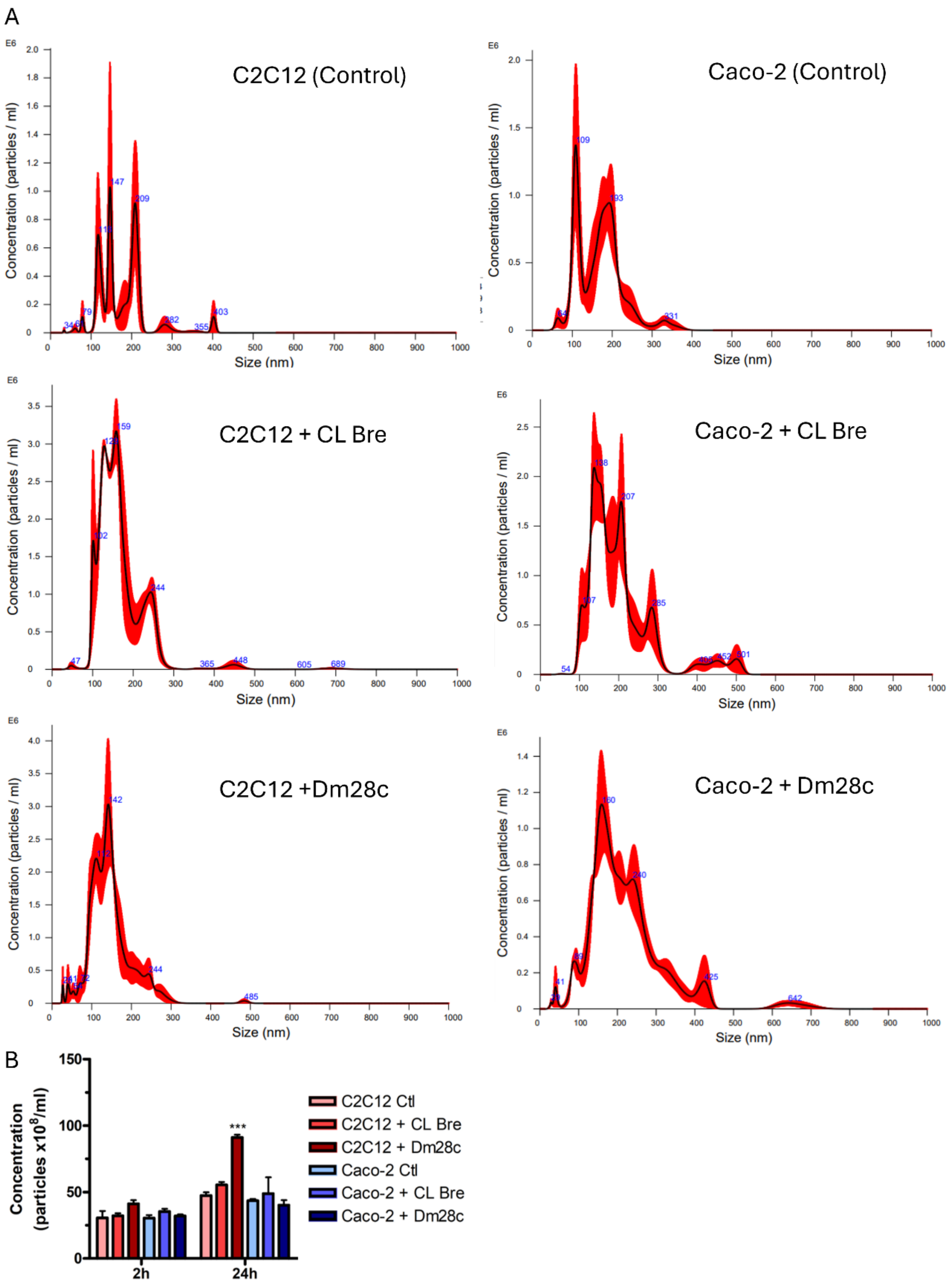

Supplementary Figure 2

Profile of LEVs secreted from control and infected cells (C2C12 and Caco-2). A) Size distribution of LEVs obtained from C2C12 and Caco-2 cells in contact with *T. cruzi*. B) Concentration of LEVs obtained from C2C12 and Caco-2 cells in contact with *T. cruzi.*

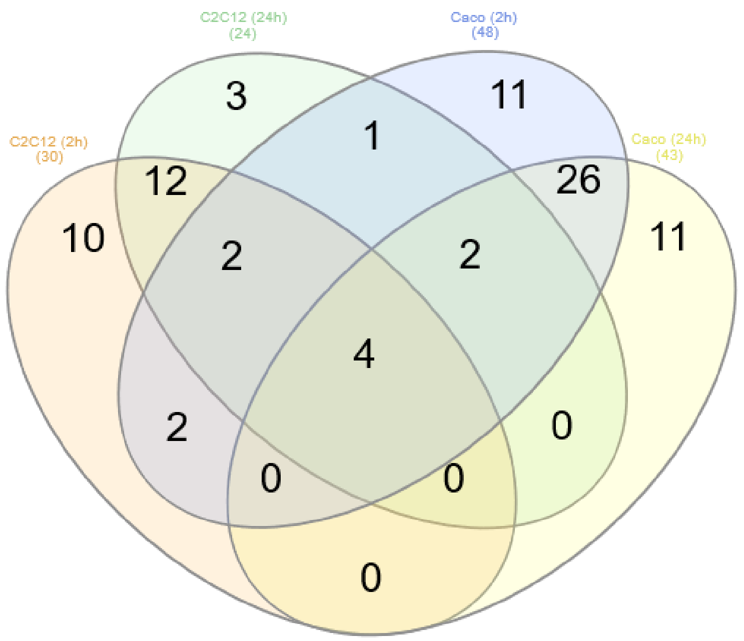

Supplementary Figure 3

Venn Diagram of proteins found in control LEVs (i.e. cells without contact with parasites for 2 and 24 hours).

TABLES:

Supplementary Table 1

Listo of proteins found in LEVs (2 h and 24 h) derived from C2C12 and Caco-2.

Supplementary Table 2

Subcellular localization of proteins found in LEVs (2 h and 24 h).

Supplementary Table 3

GO enrichment analysis of proteins found in LEVs

Supplementary Table 4

Protein-protein interaction analysis of proteins found in LEVs (STRING).

Supplementary Table 5

Overlaped proteins found in LEVs and with proteins found in EVs described by Ramirez et al. (2017).

Supplementary Table 6

IBAQ values of proteins found in LEVs derived from C2C12 and Caco-2 (2 h and 24 h).
